# Type 2 diabetes, metabolic health, and the development of frozen shoulder: a cohort study in UK electronic health records

**DOI:** 10.1186/s12891-025-08672-2

**Published:** 2025-05-14

**Authors:** Brett P. Dyer, Claire Burton, Trishna Rathod-Mistry, Miliça Blagojevic-Bucknall, Danielle A. van der Windt

**Affiliations:** 1 Griffith Biostatistics Unit, Griffith Health, Griffith University, Gold Coast, Queensland Australia; 2 Primary Care Centre Versus Arthritis, School of Medicine, Keele University, Staffordshire, UK; 3 Pharmaco- and Device Epidemiology Group, Centre for Statistics in Medicine, Nuffield Department of Orthopaedics, Rheumatology and Musculoskeletal Sciences, University of Oxford, Oxford, UK

**Keywords:** Adhesive capsulitis, Diabetes, Frozen shoulder, Mediation analysis, Metabolic syndrome

## Abstract

**Objective:**

Estimate the effect of type 2 diabetes on the development of frozen shoulder and investigate whether the effect is mediated by other metabolic factors.

**Methods:**

Primary care medical record-based cohort study containing 43,977 people newly diagnosed with type 2 diabetes and 43,977 without diabetes. Variables were identified using established Read codes. A weighting approach with Cox regression was used to decompose the total effect into the direct effect and indirect effect, mediated by metabolic health (which was defined as the number of metabolic factors developed during follow-up). Estimates were expressed as hazard ratios (HR). Confounders were identified using a DAG. Sensitivity to unmeasured confounding, extreme weights, and missing data were tested.

**Results:**

The total effect of type 2 diabetes on the development of frozen shoulder was HR = 4.38 (95% CI: 3.70–5.21), the natural indirect effect (mediated through metabolic health) was HR = 0.98 (95% CI: 0.93–1.03) and the natural direct effect was HR = 4.46 (95% CI: 3.68–5.41). Results were robust to unmeasured confounding, extreme weights, and missing data.

**Conclusions:**

This study suggests that type 2 diabetes may be a cause of frozen shoulder but does not support the hypothesis that the effect is mediated by metabolic health. Clinicians should remain alert that shoulder pain in people with diabetes could be indicative of a frozen shoulder. This study should raise awareness that, despite often being overlooked, musculoskeletal conditions can be complications of diabetes and should be considered during clinical conversations with patients.

**ISAC protocol registration number:**

19_219R.

**Supplementary Information:**

The online version contains supplementary material available at 10.1186/s12891-025-08672-2.

## Introduction

### Background

Frozen shoulder is a common, painful condition that restricts shoulder function and inhibits the patient’s ability to carry out basic everyday tasks [1, 2]. The condition arises from the contracture of the glenohumeral joint capsule, although the exact underlying aetiology is unclear [3–5]. Current histological evidence suggests that the pathogenesis may start with an inflammatory process which leads to capsular fibrosis [6].

It has been hypothesised that diabetes may play a role in initiating the aforementioned inflammatory-fibrotic process [7, 8]. Diabetes (types 1 and 2) is the most common comorbidity observed in people with frozen shoulder, with a prevalence of 30% [9]. A systematic review including two cohort studies and six case-control studies found evidence to suggest that diabetes is associated with the onset of frozen shoulder [10]. Since the publication of the review, an additional cohort study [11] and four case-control studies [12–15] have also found an association. However, limited numbers of cohort studies and inadequate adjustment for confounding variables reduces the current certainty in evidence of whether diabetes is a potential cause of frozen shoulder [10]. The first objective of this cohort study was to address the gaps in existing evidence by using causal inference methods, with the aim of estimating the causal effect of type 2 diabetes on the development of frozen shoulder.

There are currently two, not necessarily mutually exclusive, hypotheses for the reasons why diabetes may cause frozen shoulder. Firstly, hyperglycaemia promotes glycation of capsule tissues, resulting in collagen cross-linking which would explain the observed capsular fibrosis in frozen shoulder [16]. Secondly, the inflammation associated with diabetes could initiate the inflammatory-fibrotic process underlying the pathogenesis of frozen shoulder [7, 8]. Some authors have suggested that inflammation associated with metabolic syndrome, not just type 2 diabetes, could initiate the development of frozen shoulder and requires more research [7, 17]. The other components of metabolic syndrome (hyperlipidaemia, hypertension, obesity) have been shown to be prevalent amongst people with frozen shoulder [18–20], but longitudinal analysis of the association between metabolic syndrome and frozen shoulder is scarce. The call for more research on the association between metabolic syndrome and frozen shoulder motivated the second objective of this study, which was to investigate whether the increased inflammation associated with metabolic syndrome could mediate the effect of type 2 diabetes on frozen shoulder.

### Objectives

Objective 1 – Estimate the causal effect of type 2 diabetes on the development of frozen shoulder.

Objective 2 – Estimate the proportion of the effect of type 2 diabetes on the risk of developing frozen shoulder that is mediated by metabolic health.

## Methods

### Study design, setting and participants

This population-based matched cohort study was conducted using UK primary care electronic health records from the Clinical Practice Research Datalink (CPRD) GOLD database [21]. In February 2020, CPRD GOLD contained patient records for 18.5 million people [22]. The CPRD GOLD population’s age, gender, and ethnicity distributions are broadly representative of the UK population [21]. To obtain deprivation and ethnicity data, CPRD data were linked to Index of Multiple Deprivation (IMD) data and Hospital Episode Statistics (HES) data [21, 23]. The start date for this study (1st May 2004) was set to coincide with the introduction of the Quality Outcomes Framework which improved the quality of primary care coding, especially for people with diabetes [24, 25].

People were eligible for this study if they were aged 18 years or older and had their first ever Read code for type 2 diabetes between 1st May 2004 and 31st December 2017. Each individual’s index date was defined as the date of their first ever type 2 diabetes Read code. Each person with type 2 diabetes was matched on exact age, gender, and practice to a single individual without a diabetes Read code prior to the index date of their matched pair. The people without diabetes needed to be alive and registered with a CPRD practice on the index date. People were ineligible if they had any shoulder pain Read codes prior to the index date. It was also required that all people had at least two years of “up-to-standard” [21] data on the index date. People were followed up until the earliest of: end of the study follow-up period (17th February 2020); date of death (derived from CPRD data); date of last data collection from their practice.

This study was approved by the Independent Scientific Advisory Committee for Medicines and Healthcare products Regulatory Agency (19_219R).

This study did not involve patients or the public in the design, conduct, or reporting, or dissemination plans of our research.

### Variables

**Fig. 1:**
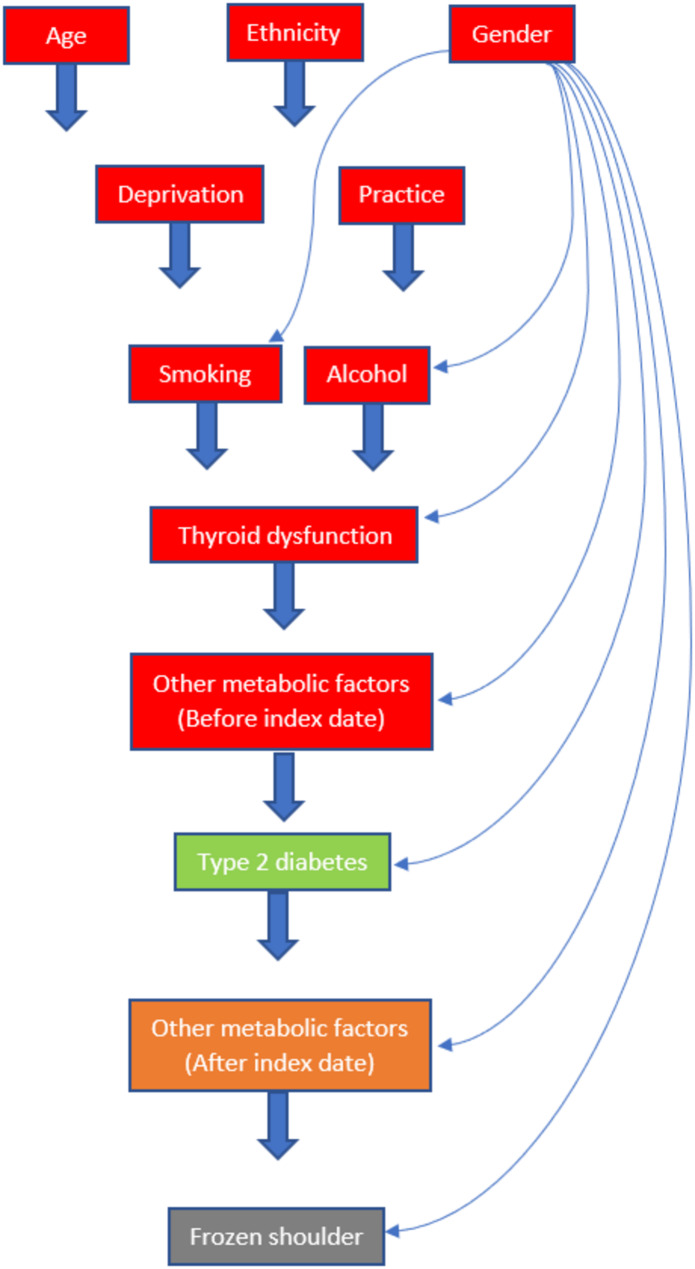
A DAG to illustrate plausible relationships between covariates, type 2 diabetes, metabolic factors, and frozen shoulder. Block arrows represent arcs to all nodes below. (This decision was made to avoid overcrowding the diagram.) Thin arrows represent potential direct causal relationships between variables. The exposure, type 2 diabetes, is green; the outcome, frozen shoulder, is grey; the mediator, other metabolic factors (post-index date), is orange; exposure-outcome confounders are red. All exposure-outcome confounders were also mediator-outcome confounders (and vice versa). Note that it was not required to create a composite variable `number of metabolic factors’ for pre-index date metabolic factors to meet the confounding assumptions; thus, pre-index date hyperlipaemia, hypertension, and obesity are left as separate variables. The variables are represented by one node, ``Other metabolic factors (Before index date)’’, in the DAG to avoid overcrowding. The only omitted arcs are from gender to deprivation and from gender to practice; this can be argued since geographical areas and practices will be unlikely to significantly differ from a 50/50 gender split

To assess whether the effect of type 2 diabetes on the development of frozen shoulder was mediated by metabolic health, the mediator was defined as the number of metabolic factors (hyperlipidaemia, hypertension, obesity) developed during follow-up (post-index date). The mediator was classed as either 0, 1, or ≥ 2 metabolic factors developed since only a small proportion of people developed all 3 metabolic factors during the follow-up period (Table 1). The role of confounders was visualised using a Directed Acyclic Graph (DAG) [26] (Fig. 1), which was constructed prior to data acquisition based on knowledge of the causal relationships between covariates, type 2 diabetes, metabolic factors, and frozen shoulder. The DAG was constructed using the guidelines provided by Tennant et al. [27]. All variables were defined using Read Code lists which were either taken from peer-reviewed publications or were constructed by an academic GP with experience of using electronic health records for research. Read code lists and their method of construction are provided in Appendix A. The factor levels for categorical variables (type 2 diabetes, frozen shoulder, hyperlipidaemia, hypertension, obesity, ethnicity, alcohol, smoking, gender, deprivation, and thyroid dysfunction) are given in Appendix B.

**Table 1:** Table of baseline characteristics for participants with type 2 diabetes and participants without diabetes. 1 defined as time from index date to the earliest of: end of study (17th February 2020), date of death, date of transfer to a non-CPRD practice, or date of last CPRD data collection. 2 hyperlipidaemia, hypertension, or obesity. 3 referring to other non-specific codes that could relate to hypothyroidism or hyperthyroidism

	Type 2 diabetes	No diabetes	All
	*n* = 43,977 (50%)	*n* = 43,977 (50%)	*n* = 87,954
**Median follow-up** ^**1**^ **in years (IQR)**	8.24 (4.90–11.65)	9.06 (5.95–12.19)	8.69 (5.42–11.93)
**Mean age (years)**	59.43 (SD = 14.01)	59.43 (SD = 14.01)	59.43 (SD = 14.01)
**Gender**			
Male	25,236 (57.38%)	25,236 (57.38%)	50,472 (57.38%)
Female	18,741 (42.62%)	18,741 (42.62%)	37,482 (42.62%)
**Hypertension**			
**(pre-index date)**			
Diagnosed	21,538 (48.98%)	9,803 (22.29%)	31,341 (35.63%)
Not diagnosed	22,439 (51.02%)	34,174 (77.71%)	56,613 (64.37%)
**Hyperlipidaemia**			
**(pre-index date)**			
Diagnosed	8,649 (19.67%)	3,782 (8.60%)	12,431 (14.13%)
Not diagnosed	35,328 (80.33%)	40,195 (91.40%)	75,523 (85.87%)
**Obesity**			
**(pre-index date)**			
Obese	23,603 (53.77%)	6,687 (15.23%)	30,290 (34.50%)
Not obese	17,599 (40.09%)	25,847 (58.88%)	43,446 (49.49%)
Missing	2,693 (6.14%)	11,361 (25.88%)	14,054 (16.01%)
**Number of metabolic factors** ^**2**^ **developed during follow-up**			
0	30,542 (69.45%)	38,609 (87.79%)	69,151 (78.62%)
1	11,052 (25.13%)	4,466 (10.16%)	15,518 (17.61%)
2	2,173 (4.94%)	828 (1.88%)	3,001 (3.41%)
3	210 (0.48%)	74 (0.17%)	284 (0.32%)
**Thyroid dysfunction**			
Diagnosed	4,014 (9.13%)	2,396 (5.45%)	6,410 (7.29%)
Not diagnosed	39,963 (90.87%)	41,581 (94.55%)	81,544 (92.71%)
**Type of thyroid dysfunction Read code**			
Congenital	13 (0.03%)	7 (0.02%)	20 (0.02%)
Hyperthyroidism	625 (1.42%)	418 (0.95%)	1,043 (1.19%)
Hypo/Hyperthyroidism^3^	588 (1.34%)	319 (0.73%)	907 (1.03%)
Hypothyroidism	3,043 (6.92%)	1,622 (3.69%)	4,665 (5.30%)
Malignant	17 (0.04%)	19 (0.04%)	36 (0.04%)
Surgery	398 (0.91%)	287 (0.65%)	685 (0.78%)
Other	1,010 (2.30%)	703 (1.60%)	1,713 (1.95%)
**Smoking**			
Yes	8,149 (18.53%)	8,498 (19.32%)	16,647 (18.93%)
No	20,686 (47.04%)	20,292 (46.14%)	40,978 (46.59%)
Ex	14,398 (32.74%)	8,599 (19.55%)	22,997 (26.15%)
Missing	744 (1.69%)	6,588 (14.98%)	7,332 (8.34%)
**Alcohol**			
Yes	29,468 (67.01%)	27,074 (61.56%)	56,542 (64.29%)
No	8,090 (18.40%)	4,814 (10.95%)	12,904 (14.67%)
Ex	1,487 (3.38%)	584 (1.33%)	2,071 (2.35%)
Missing	4,932 (11.21%)	11,505 (26.16%)	16,437 (18.69%)
**Ethnicity**			
Bangladeshi	124 (0.28%)	47 (0.11%)	171 (0.19%)
Black African	184 (0.42%)	102 (0.23%)	286 (0.33%)
Black Caribbean	223 (0.51%)	141 (0.32%)	364 (0.41%)
Black – other	78 (0.18%)	59 (0.13%)	137 (0.16%)
Chinese	79 (0.18%)	56 (0.13%)	135 (0.15%)
Indian	590 (1.34%)	184 (0.42%)	774 (0.88%)
Mixed	168 (0.38%)	93 (0.21%)	261 (0.30%)
Other Asian	316 (0.72%)	98 (0.22%)	414 (0.47%)
Other	356 (0.81%)	286 (0.65%)	642 (0.73%)
Pakistani	320 (0.73%)	141 (0.32%)	461 (0.52%)
Missing	11,020 (25.06%)	12,331 (28.04%)	23,351 (26.55%)
White	30,519 (69.40%)	30,439 (69.22%)	60,958 (69.31%)
**IMD Quintile**			
Least deprived quintile	8,535 (19.41%)	9,899 (22.51%)	18,434 (20.96%)
2nd least deprived quintile	9,178 (20.87%)	9,675 (22.00%)	18,853 (21.44%)
3rd least deprived quintile	9,376 (21.32%)	9,320 (21.19%)	18,696 (21.26%)
4th least deprived quintile	8,811 (20.04%)	8,140 (18.51%)	16,951 (19.27%)
Most deprived quintile	8,052 (18.31%)	6,885 (15.66%)	14,937 (16.98%)
Missing	25 (0.06%)	58 (0.13%)	83 (0.09%)

### Statistical methods

A weighting approach [28–30] was used to estimate the natural direct and natural indirect effects with a Cox proportional hazards model. For a binary exposure, the natural direct effect is the effect of the exposure on the outcome whilst fixing the mediator to the value it would have been had the person not been exposed. The natural indirect effect is the effect of changing the mediator from the value it would have taken in the absence of the exposure to the value it would take in the presence of the exposure while keeping the exposure fixed to being exposed.

The weighted approach to causal mediation analysis, set in the counterfactual framework, uses an exposure weight (a stabilised inverse probability weight) with the aim of creating a pseudo-population in which the value that the exposure takes should be independent of the value of the set of confounders (attempting to mimic a randomised trial). The exposure weighting aims to account for exposure-mediator confounding and exposure-outcome confounding; however, mediator-outcome confounding also needs to be accounted for in a causal mediation analysis. A second weighting method, called Ratio of Mediator Probability Weighting (RMPW) [29, 30] was used to decompose the total effect into the natural direct effect and the natural indirect effect while accounting for mediator-outcome confounding. After the two weighting approaches have been applied, a Cox proportional hazards model can be used, and the two Cox model coefficients provide the estimates of the natural direct effect and natural indirect effect on the log hazard ratio scale. The total effect hazard ratio is given by the product of the natural direct effect hazard ratio and natural indirect effect hazard ratio. Bootstrapping must be used to obtain 95% confidence intervals [31]. The presence of exposure-mediator interaction was tested.

Causal mediation analysis requires the following no unmeasured confounding assumptions: no unmeasured exposure-outcome confounding, no unmeasured mediator-outcome confounding, no unmeasured exposure-mediator confounding, no mediator-outcome confounder is caused by the exposure [32]. The DAG implied adjustment set was: age; gender; ethnicity; deprivation; practice; smoking; alcohol thyroid dysfunction; and pre-index date hyperlipidaemia, hypertension, and obesity.

Mediator weights were obtained through ordinal logistic regression as the mediator, the number of metabolic factors developed during follow-up, is ordinal. Exposure weights were obtained through binary logistic regression as the exposure, type 2 diabetes, is binary. This was a matched cohort study, so generalised estimating equations were used in the ordinal logistic regression and binary logistic regression models to account for the lack of independence between matched individuals. Individuals were censored upon death, transfer to a non-CPRD practice, at the end of follow-up (17th February 2020), or if the person obtained their first-ever type 2 diabetes Read code after the index date. The proportional hazards assumption was assessed using Schoenfeld residual plots. The missing indicator method (use of a dummy variable to indicate missingness on a particular variable) was used to handle missing data for smoking, alcohol, ethnicity, and obesity; otherwise, it was assumed that the absence of a Read code indicated the absence of the disease. A Kaplan-Meier plot and estimates for not being diagnosed with a frozen shoulder were also presented.

Data were prepared in Stata version 14.0 and analysed in RStudio version 1.2.5033.

### Sensitivity analyses

An E-value [33] was calculated to assess the strength of unmeasured confounding that would be required to completely explain away an estimated effect. The Cox model was re-run with truncated weights (exposure weights exceeding the 95th percentile were truncated at the value of the 95th percentile) to assess the sensitivity of results to extreme weights. A complete case analysis was conducted to assess the sensitivity of results to missing data.

## Results

### Sample characteristics

The study contained 43,977 people newly diagnosed with type 2 diabetes matched to 43,977 people without diabetes (Table 1). 57% of the people with type 2 diabetes were male, and the mean age at diagnosis was 59.4 years (SD = 14.0). The median follow-up^1^ was 8.2 years (IQR: 4.9–11.7) for the type 2 diabetes group and 9.1 years (IQR: 6.0–12.2) for the group without diabetes. The individuals with type 2 diabetes were less likely to have missing data for their alcohol and smoking statuses. (Note that people who require routine health monitoring are more likely to have complete alcohol and smoking records [34], which would indicate data are missing not at random.)

### Results for the effect of type 2 diabetes on the development of frozen shoulder

**Fig. 2:**
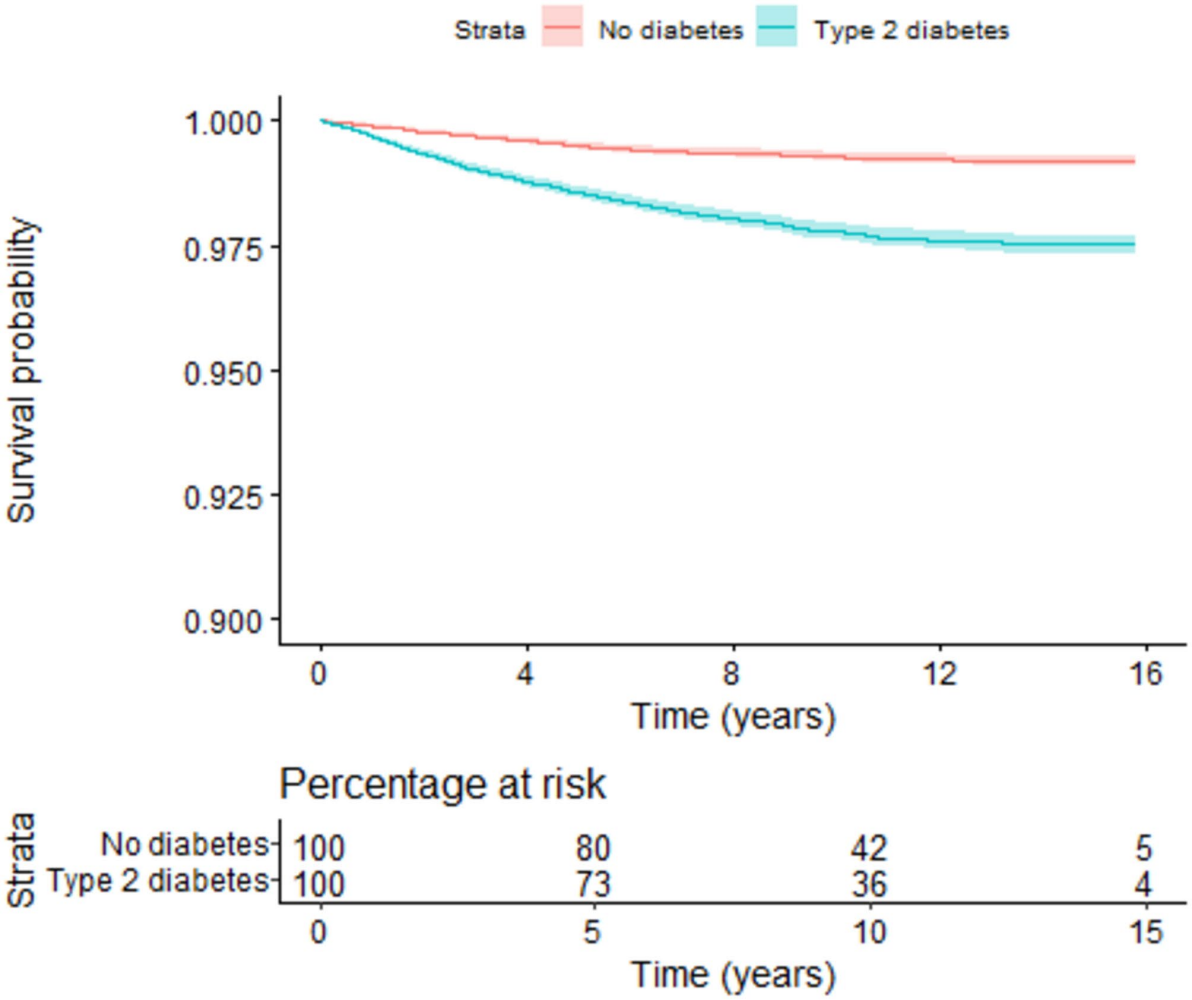
A plot of Kaplan-Meier estimates (with 95% confidence intervals) for not being diagnosed with a frozen shoulder in people with type 2 diabetes versus people without diabetes, including a percentage at risk table

During follow-up, 1076 (1.22%) individuals developed frozen shoulder. Within the people with diabetes, 797 (1.81%) people developed frozen shoulder; and within the people without diabetes, 279 (0.63%) people developed frozen shoulder. The 1-, 3-, 5-, 10-, and 15.8-year Kaplan-Meier estimates for not being diagnosed with a frozen shoulder were 99.7%, 99%, 98.6%, 97.8%, 97.7% for people with diabetes and > 99.9%, 99.7%, 99.5%, 99.3%, 99.3% for people without diabetes (Fig. 2).

The estimate for the total effect of type 2 diabetes on the development of frozen shoulder was HR = 4.38 (95% CI: 3.70–5.21). The E-value, an estimate of the strength of effect that a hypothetical unmeasured confounder would need to have on the exposure and outcome to completely explain away the effect estimate, was 4.87 for the point estimate and 4.30 for the 95% CI lower bound. This means that it would require an unmeasured confounder that is strongly associated with both type 2 diabetes and frozen shoulder in order to completely explain away the total effect estimate.

The natural indirect effect of type 2 diabetes on the development of frozen shoulder that is mediated through metabolic health was estimated to be HR = 0.98 (95% CI: 0.93–1.03) and the remaining natural direct effect was estimated to be HR = 4.46 (95% CI: 3.68–5.41). Thus, there was no evidence that metabolic health mediated the effect of type 2 diabetes on the development of frozen shoulder. The exposure-mediator interaction term was excluded since there was no evidence of interaction (HR = 0.98). There was no evidence to suggest any violation of the proportional hazards assumptions (Fig. 2, Figures C1 and C2).

When repeating the analysis with truncated weights, the results were similar. The total effect was HR = 3.10 (95% CI: 2.78–4.13), the natural indirect effect was HR = 0.93 (95% CI: 0.90–1.07), and the natural direct effect was HR = 3.32 (95% CI: 2.90–4.15). Thus, the results were robust to extreme weights. The distribution of exposure weights ($\:{W}_{i}^{X}$), mediator weights ($\:{W}_{i}^{M}$), final untruncated model weights ($\:{W}_{i},\text{untruncated}$ = $\:{W}_{i}^{X}.\:{W}_{i}^{M}$), and final truncated model weights ($\:{W}_{i},\text{truncated}$) are shown in Figures D1-D4.

Conducting a complete case analysis (80.5% of people had complete data) also produced similar results, but with a smaller direct effect (and therefore also a small total effect). The total effect was HR = 2.59 (95% CI: 2.20–3.05), the natural indirect effect was HR = 0.99 (95% CI: 0.94–1.04), and the natural direct effect was HR = 2.62 (95% CI: 2.21–3.09). So, the hazard ratio was smaller in magnitude when only those with complete data were included in the analysis, but the estimated causal effect was still strong. Thus, regardless of how missing data was handled, the conclusion drawn is the same - the results suggest a causal effect of type 2 diabetes on the development of frozen shoulder.

## Discussion

This study has found evidence to support the hypothesis that type 2 diabetes may be a cause of frozen shoulder. It had previously been suggested that the inflammation associated with type 2 diabetes and metabolic health may be an important factor in the development of frozen shoulder [7, 17]; although, the results of this causal mediation analysis did not support the hypothesis that the effect of type 2 diabetes on the development of frozen shoulder is mediated by metabolic health. The large E-value suggested that it would require an unmeasured confounder with a strong effect on both type 2 diabetes and frozen shoulder to completely explain away the total effect estimate; thus, results were robust to unmeasured confounding. Sensitivity analyses also suggested that the results were also robust to missing data and extreme weights.

To conduct the mediation analysis, it was necessary to classify the mediator as `the number of metabolic factors developed during follow-up’. It was not possible to investigate the separate metabolic factors as individual mediators since they would be mediator-outcome confounders for each other and are all caused by the exposure; thus, the no unmeasured confounding assumptions would be violated. Also, it was opted to include “the number of metabolic factors developed” as the mediator rather than a binary variable indicating whether the individual had metabolic syndrome since this provided more information about the person’s metabolic health (e.g., an individual that has two metabolic factors, but not metabolic syndrome, will have worse metabolic health than an individual without any metabolic factors).

The number of metabolic factors developed during follow-up is likely to be a good indicator of an individual’s metabolic health; however, this approach does have limitations. This composite variable treats each individual metabolic factor equally, such that an individual who developed hypertension and an individual who developed hyperlipidaemia would both be classed in the category ``one metabolic factor developed’’. Further, this way of classifying metabolic health requires that the metabolic factors be dichotomised, which also leads to loss of information. Given that there are limitations in the way that metabolic health was defined, the results of this study mustn’t be used to completely reject the hypothesis that metabolic health could mediate the effect of type 2 diabetes on the development of frozen shoulder; rather, the results have failed to support the hypothesis.

Read codes were used to identify the outcome in this study. At the end of follow-up, the Kaplan-Meier estimate for being coded as having developed frozen shoulder within the individuals with type 2 diabetes was 2.3%. This is much less than expected given that the prevalence of frozen shoulder in people with diabetes is estimated to be 13.4%. Research has shown that general practitioners (GPs) in the UK and the Netherlands are hesitant to give specific shoulder condition diagnoses but prefer to use non-specific shoulder pain codes [35, 36]. Further, the pre-existing knowledge of the association between diabetes and frozen shoulder may mean that GPs are more likely to give a specific frozen shoulder diagnosis to a person with diabetes than a person without diabetes. Thus, this may have led to some overestimation of association sizes.

To our knowledge, this is the first time this approach to causal mediation analysis has been conducted using data coming entirely from electronic health records. Using electronic health records provided a large, representative sample of people with type 2 diabetes; although, a limitation is that there are large amounts of missing data [21]. The missing indicator method is commonly used to handle missing data in the analysis of medical records to avoid losing a large proportion of participants and losing the representativeness of the sample. The missing indicator method can produce biased estimates; however, there are a lack of alternatives since multiple imputation methods are inappropriate when data are missing not at random, which is the case for BMI, ethnicity, smoking, and alcohol data [37]. A complete case analysis was conducted to assess the sensitivity of results to missing data and suggested that the results were robust.

Previously, three cohort studies [11, 38, 39] had found evidence that type 2 diabetes was associated with the onset of frozen shoulder. This study was the first to use causal inference methods to estimate the effect of type 2 diabetes on frozen shoulder development and was the first study to investigate one of the proposed mechanisms of how type 2 diabetes may lead to frozen shoulder. Further, this is the first cohort study addressing this question outside of East Asia. Despite differences in population, environmental, and lifestyle characteristics, and differences in health care strategies (e.g., the QOF, routine diabetes reviews and screening strategies), the conclusions of this study are consistent with the three East Asian cohort studies [11, 38, 39].

Future research should focus on investigating the other main hypothesis for how diabetes may lead to the development of frozen shoulder – that glycation processes alter shoulder capsule tissues. One study did not find evidence to suggest that HbA1c was associated with the prevalence of frozen shoulder; although, the study was limited by only including HbA1c as a single measurement within a cross-sectional analysis [40]. Another study generated a non-validated variable to indicate historical HbA1c values and found that poor glycaemic control was associated with the development of frozen shoulder [41]. To improve the understanding of this association, joint modelling strategies could be used to estimate the association between longitudinally measured HbA1c and the development of frozen shoulder using a cohort study design. This research could also include people with type 1 diabetes. To meet the causal identifiability assumptions required for this study it was necessary to only include people with type 2 diabetes, but previous research has shown type 1 diabetes to also be associated with frozen shoulder [9]. Additionally, future work could further investigate whether blood pressure, waist circumference, and lipid measures (in their non-dichotomised form) are associated with the onset frozen shoulder. This would improve the understanding of the role of metabolic health and inflammation in the development of frozen shoulder.

## Conclusion

The results of this study support the hypothesis that type 2 diabetes is a cause of frozen shoulder but did not suggest that this effect is mediated by metabolic health. Clinicians should remain alert that shoulder pain in people with type 2 diabetes could be indicative of a frozen shoulder. This study also should raise awareness that, despite often being overlooked, musculoskeletal conditions can be complications of diabetes and should be considered during clinical conversations with patients.

## Electronic supplementary material

Below is the link to the electronic supplementary material.

Supplementary Material 1

Supplementary Material 2

Supplementary Material 3

Supplementary Material 4

## Data Availability

The data that support the findings of this study are available on request from the corresponding author.

## Footnotes

1 Defined as time from index date to the earliest of: end of study (17th February 2020), date of death, date of transfer to a non-CPRD practice, or date of last CPRD data collection.

## Abbreviations

CPRD: Clinical practice research datalink
DAG: Directed acyclic graph
HES: Hospital episode statistics
HR: Hazard ratio
IMD: Index of multiple deprivation
RMPW: Ratio of mediator probability weighting

## References

[1] Robinson CM, Seah KTM, et al. Frozen shoulder. J Bone Joint Surg. 2012;94(1):1–9. 10.1302/0301-620X.94B1.27093 22219239

[2] Jones S, Hanchard N, et al. A qualitative study of patients’ perceptions and priorities when living with primary frozen shoulder. BMJ Open. 2013;3:e003452. 24078753 10.1136/bmjopen-2013-003452 PMC3787409

[3] Dias R, Cutts S, et al. Frozen Shoulder BMJ. 2005;331(7530):1453–6. 10.1136/bmj.331.7530.1453 PMC1315655 16356983

[4] Ewald A. Adhesive capsulitis: A review. Am Family Phys. 2011;83(4):417–22. 21322517

[5] Hand GCR, Athanasou NA, et al. The pathology of frozen shoulder. J Bone Joint Surg. 2007;89(7):928–32. 10.1302/0301-620X.89B7.19097 17673588

[6] Tamai K, Hamada J, et al. Frozen shoulder. An overview of pathology and biology with hopes to novel drug therapies. Mod Rheumatol. 2024;34(3):439–43. 37632764 10.1093/mr/road087

[7] Cucchi D, Marmotti A, et al. Risk factors for shoulder stiffness: current concepts. Joints. 2017;5(4):517–223. 10.1055/s-0037-1608951 PMC5738468 29270559

[8] Hsu CL. Sheu. Diabetes and shoulder disorders. J Diabetes Investig. 2016;7(5):649–51. 10.1111/jdi.12491. 27182002 10.1111/jdi.12491 PMC5009124

[9] Zreik NH, Malik RA, et al. Adhesive capsulitis of the shoulder and diabetes: a meta-analysis of prevalence. Muscle Ligament Tendons. 2016;6(1):26–34. 10.11138/mltj/2016.6.1.026 PMC4915459 27331029

[10] Dyer BP, Rathod-Mistry T, et al. Diabetes as a risk factor for the onset of frozen shoulder: a systematic review and meta-analysis. BMJ Open. 2013;13(1):e062377. 10.1136/bmjopen-2022-062377 PMC9815013 36599641

[11] Kim J-H, Kim B-S, et al. The risk of shoulder adhesive capsulitis in individuals with prediabetes and type 2 diabetes mellitus: A longitudinal nationwide Population-Based study. Diabetes Metab J. 2023;47(6):869–78. 37915186 10.4093/dmj.2022.0275 PMC10695720

[12] Sun G, Li Q, et al. Risk factors and predictive models for frozen shoulder. Sci Rep. 2024;14:15261. 38956312 10.1038/s41598-024-66360-y PMC11220144

[13] Abudula X, Maimaiti P, et al. Factors associated with frozen shoulder in adults: a retrospective study. BMC Musculoskelet Disord. 2024;25:493. 38926699 10.1186/s12891-024-07614-8 PMC11200817

[14] Yang W, Yang Y, et al. Socioeconomic status, obesity, individual behaviors, diabetes, and risk for frozen shoulder: a Mendelian randomization study. Medicine. 2023;102(49):e36470. 38065922 10.1097/MD.0000000000036470 PMC10713162

[15] Green HD, et al. A genome-wide association study identifies 5 loci associated with frozen shoulder and implicates diabetes as a causal risk factor. PLoS Genet. 2021;17(6):e1009577. 34111113 10.1371/journal.pgen.1009577 PMC8191964

[16] Hwang KR, Murrell GA, et al. Advanced glycation end products in idiopathic frozen shoulders. J Shoulder Elbow Surg. 2016;25(6):981–8. 26776943 10.1016/j.jse.2015.10.015 PMC5402873

[17] Pietrzak M. Adhesive capsulitis: an age-related symptom of metabolic syndrome and chronic low-grade inflammation? Med Hypotheses. 2016;88:12–7. 26880627 10.1016/j.mehy.2016.01.002

[18] Austin DC, Gans I, et al. The association of metabolic syndrome markers with adhesive capsulitis. J Shoulder Elbow Surg. 2014;23:1043–51. 24560465 10.1016/j.jse.2013.11.004

[19] Sung CM, Sung TS, et al. Are serum lipids involved in primary frozen shoulder? A case-control study. J Bone Joint Surg. 2014;96(21):1828–33. 25378511 10.2106/JBJS.M.00936

[20] Nayak SP. Panda. Is hyperlipidaemia a cause of primary frozen shoulder? A case-controlled study. J Evid Based Med Healthc. 2017;4(13):697–701.

[21] Herrett E, Gallagher AM, et al. Data resource profile: clinical practice research datalink (CPRD). Int J Epidemiol. 2015;44(3):827–36. 26050254 10.1093/ije/dyv098 PMC4521131

[22] Clinical Practice Research Datalink. 2021, Available from: https://www.cprd.com [accessed 17th February 2022].

[23] Padmanabhan S, Carty L, et al. Approach to record linkage of primary care data from clinical practice research datalink to other health-related patient data: overview and implications. Eur J Epidemiol. 2019;34:91–9. 30219957 10.1007/s10654-018-0442-4 PMC6325980

[24] Kontopantelis E, Reeves D, et al. Recorded quality of primary care for patients with diabetes in England before and after the introduction of a financial incentive scheme: a longitudinal observational study. BMJ quality & safety; 2013;53–64. 10.1136/bmjqs-2012-001033 22918988

[25] Khunti K, Gadsby R, et al. Quality of diabetes care in the UK: comparison of published quality-of-care reports with results of the quality and outcomes framework for diabetes. Diabet Med. 2007;24:1436–41. 17971182 10.1111/j.1464-5491.2007.02276.x

[26] Greenland S, Pearl J, et al. Causal diagrams for epidemiologic research. Epidemiology. 1999;10:37–48. 9888278

[27] Tennant PWG, Murray EJ et al. Use of directed acyclic graphs (DAGs) to identify confounders in applied health research: review and recommendations. Int J Epidemiol, 2020;50(2):620–32. 10.1093/ije/dyaa213 PMC8128477 33330936

[28] Lange T, Vansteelandt S, et al. A simple unified approach for estimating natural direct and indirect effects. Am J Epidemiol. 2012;176(3):190–5. 22781427 10.1093/aje/kwr525

[29] Hong G. Ratio of mediator probability weighting for estimating natural direct and indirect effects. JSM Proceedings, Biometrics Section, 2010;2401–2415.

[30] Hong G, Deutsch J, et al. Ratio-of-mediator-probability weighting for causal mediation analysis in the presence of treatment-by-mediator interaction. J Educational Behav Stat. 2015;40(3):307–40.

[31] T. J. Vander Weele. Explanation in causal inference. Oxford University Press, 2015.

[32] Vander Weele TJ. Mediation analysis: A practitioner’s guide. Annu Rev Public Health. 2016;37:17–32. 26653405 10.1146/annurev-publhealth-032315-021402

[33] VanderWeele TJ, Peng D. Sensitivity analysis in observational research: introducing the E-value. Ann Intern Med. 2017;167(4):268–74. 28693043 10.7326/M16-2607

[34] Mansfield K, Crellin E, et al. Completeness and validity of alcohol recording in general practice within the UK: a cross-sectional study. BMJ Open. 2019;9:e031537. 31772094 10.1136/bmjopen-2019-031537 PMC6887039

[35] Linsell L, Dawson J, et al. Prevalence and incidence of adults consulting for shoulder conditions in UK primary care; patterns of diagnosis and referral. Rheumatology. 2006;45:215–21. 16263781 10.1093/rheumatology/kei139

[36] Dorrestijn O, Greving K. Patients with shoulder complaints in general practice: consumption of medical care. Rheumatology. 2011;50(2):389–95. 21047806 10.1093/rheumatology/keq333

[37] Choi J, Dekkers OM, et al. A comparison of different methods to handle missing data in the context of propensity score analysis. Eur J Epidemiol. 2019;34:23–36. 30341708 10.1007/s10654-018-0447-z PMC6325992

[38] Lo S-F, Chu S-W, et al. Diabetes mellitus and accompanying hyperlipidemia are independent risk factors for adhesive capsulitis: a nationwide population-based cohort study. Rheumatol Int. 2014;34:67–74. 23949624 10.1007/s00296-013-2847-4

[39] Huang Y-P, Fann C-Y, et al. Association of diabetes mellitus with the risk of developing adhesive capsulitis of the shoulder: A longitudinal population-based follow-up study. Arthritis Care Res. 2013;65(7):1197–202. 10.1002/acr.21938 23281342

[40] Yian H, Contreras R, et al. Effects of glycemic control on prevalence of diabetic frozen shoulder. J Bone Joint Surg. 2012;94(10):919–23. 22617920 10.2106/JBJS.J.01930

[41] Chan JH, Ho BS, et al. The relationship between the incidence of adhesive capsulitis and haemoglobin A1c. J Shoulder Elb Surg. 2017;26(10):1834–7. 10.1016/j.jse.2017.03.015 28495575

