# Supplementary material for: Type 2 diabetes, metabolic health, and the development of frozen shoulder: a cohort study in UK electronic health records
Source: BMC Musculoskelet Disord. 2025 May 14;26:471. doi: 10.1186/s12891-025-08672-2 (PMC12080057; doi:10.1186/s12891-025-08672-2)
Supplement: Supplementary file 1 — Supplementary Material 1 [file 12891_2025_8672_MOESM1_ESM.docx]

**Appendix A – Variable Read code lists**

The type 2 diabetes Read code list in Table 2 was constructed by two general practitioners. The codes are recorded in the clinical file within CPRD GOLD.

| **Table 2** Table of type 2 diabetes Read codes. Note that “..” represents the inclusion of all daughter codes | |
| --- | --- |
| **Read code** | **Detail** |
| 66A.. | Diabetes monitoring |
| 9OL.. | Diabetic monitoring admin |
| C10 | Diabetes mellitus |
| C100 | Diab.mell. - no complication |
| C1000 | Diab.mell.no comp. - juvenile |
| C1000-1 | Insulin dependent diab mellit. |
| C1001 | Diab.mell.no comp. - adult |
| C1001-1 | Maturity onset diabetes |
| C1001-2 | Non-insulin depend.diabet.mell |
| C100z | Diab.mell.no comp. - onset NOS |
| C102 | Diab.mell. + hyperosmolar coma |
| C104 | Diabetic nephropathy with renal manifestation |
| C1041 | Diab.mell.+nephropathy - adult |
| C104-1 | Diabetic nephropathy |
| C104z | Diab.mell.+nephropathy NOS |
| C105 | Diab.mell.+ eye manifestation |
| C1051 | Diab.mell.+eye manif - adult |
| C106 | Diabetes + neuropathy |
| C106-2 | Diab.mell. with neuropathy |
| C1061 | Diab.mell.+neuropathy - adult |
| C106-1 | Diabetic amyotrophy |
| C106-3 | Diabetic mellitus with polyneuropathy |
| C106-99 | Diabetes + neuropathy |
| C106y | Oth specf diab mel+neuro comps |
| C106z | Diab.mell.+neuropathy NOS |
| C1071 | Diab.+periph.circ.dis.-adult |
| C107 | Diab.mell. With peripheral circulatory disorder |
| C107-1 | Diab.mell. With gangrene |
| C107-2 | Diab. With gangrene |
| C1074 | NIDDM periph circulat disord |
| C107z | Diab.+periph.circ.disease NOS |
| C108 | IDDM-Insul depend diabet melit |
| C108-1 | IDDM-Insul depend diabet melit |
| C1085 | Insul depen diab mel+ulcer |
| C1085-2 | Insul depen diab mel+ulcer |
| C1088 | Insul dep diab mell-poor contr |
| C109 | Non-insulin dependent diabetes mellitus |
| C1091 | Non-ins-dp diab mel+ophth comp |
| C109-1 | NIDDM - Non-insulin dependent diabetes mellitus |
| C1091-1 | Type II diab mel+ophth comp |
| C1091-2 | Type 2 diab mel+ophth comp |
| C109-2 | Type 2 diabetes mellitus |
| C1092-2 | Type 2 diab mell neurol comp |
| C109-3 | Type II diabetes mellitus |
| C1094 | Non insulin dependent diab mell with ulcer |
| C1094-1 | Type II diab mell with ulcer |
| C1094-2 | Type 2 diab mell with ulcer |
| C1097 | Non-insul dep diab-poor contr |
| C1097-1 | Type II diab-poor contr |
| C1097-2 | Type 2 diab-poor contr |
| C1099 | Non-insul-dep diab mel no comp |
| C1099-1 | Type II diabetes mellitus without complication |
| C1099-2 | Type 2 diabetes mellitus without complication |
| C109C | Non inslulin dependant diab mell nephropathy |
| C109C-1 | Type II diab mell nephropathy |
| C109C-2 | Type 2 diab mell nephropathy |
| C109J | Insul treated Type 2 diab mell |
| C109J-1 | Insul treated non-insulin dep diab mell |
| C109J-2 | Insul treated Type 2 diab mell |
| C10B | Diabet mel induced by steroids |
| C10B0 | Sterod ind diab mel w/out comp |
| C10E | Insulin dep diabetes mellitus |
| C10E-1 | Insulin dep diabetes mellitus |
| C10E-2 | Insulin dep diabetes mellitus |
| C10E8 | Insul dep diab mell-poor contr |
| C10EC-2 | Insulin dependent diabetes mellitus with polyneuropathy |
| C10F | Type 2 diabetes mellitus |
| C10F0 | Type 2 diab mell + renal compl |
| C10F0-1 | Type 2 diab mell + renal compl |
| C10F1 | Type 2 diab mell+ophthal comp |
| C10F-1 | Type II diabetes mellitus |
| C10F1-1 | Type II diab mell+ophthal comp |
| C10F2 | Type 2 diab mell + neurol comp |
| C10F2-1 | Type 2 diab mell + neurol comp |
| C10F6 | Type 2 diab mell + retinopathy |
| C10F6-1 | Type II diab mell + retinopathy |
| C10F7 | Type 2 diab mell+poor control |
| C10F7-1 | Type II diab mell+poor control |
| C10F8 | Reaven’s syndrome |
| C10F9 | Type 2 diab mell without comp |
| C10F9-1 | Type II diab mell without comp |
| C10FB | Type 2 diab mell + polyneurop |
| C10FB-1 | Type II diab mell + polyneurop |
| C10FJ | Insul treated Type 2 diab mell |
| C10FJ-1 | Insul treated Type 2 diab mell |
| C10FK | Hyperos non-ket stat typ 2 d m |
| C10FK-1 | Hyperos non-ket stat typ 2 d m |
| C10FL | Type 2 d m + persist proteinur |
| C10FL-1 | Type II d m + persist proteinur |
| C10FM | Type 2 d m + persist microalb |
| C10FM-1 | Type II d m + persist microalb |
| C10FQ | Type 2 d m + exudat maculopath |
| C10FQ-1 | Type II d m + exudat maculopath |
| C10FR | Type 2 dm with gastroparesis |
| C10y | Diab.mell.+other manifestation |
| C10zz | Diab.mell. + unspec comp NOS |

The diabetes Read code list in Table 3 was constructed by two general practitioners. The codes are recorded in the clinical file within CPRD GOLD.

| **Table 3** Table of diabetes (all types) Read codes. Note that “..” represents the inclusion of all daughter codes | |
| --- | --- |
| **Read code** | **Detail** |
| 66A | Diabetic monitoring |
| 66A1 | Initial diabetic assessment |
| 66A2 | Follow-up diabetic assessment |
| 66A3 | Diabetic on diet only |
| 66A4 | Diabetic on oral treatment |
| 66A5 | Diabetic on insulin |
| 66A6 | Last hypo. attack |
| 66A71 | Frequency GP/param hypoglycaem |
| 66A8 | Has seen dietician - diabetes |
| 66A9 | Understands diet - diabetes |
| 66AA | Injection sites |
| 66AA-1 | Injection sites - diabetic |
| 66Ab | Diabetic foot examination |
| 66Ac | Diabetic periph neurop screen |
| 66AD | Fundoscopy - diabetic check |
| 66Ae | HBA1c target |
| 66Ae0 | HBA1c target level - IFCC standardised |
| 66AG | Diabetic drug side effects |
| 66AH | Diabetic treatment changed |
| 66AH0 | Conversion to insulin |
| 66AI | Diabetic - good control |
| 66AJ | Diabetic - poor control |
| 66AJ0 | Chronic hyperglycaemia |
| 66AJ1 | Brittle diabetes |
| 66AJ-1 | Unstable diabetes |
| 66AJz | Diabetic - poor control NOS |
| 66Am | Insulin dose changed |
| 66AM | Diabetic - follow-up default |
| 66Ao | Diabetes type 2 review |
| 66Aq | Diabetic foot screen |
| 66AQ | Diabetes: shared care programme |
| 66AQ0 | Unsuit diab year care program |
| 66AR | Diabetes management plan given |
| 66AS | Diabetic annual review |
| 66AS0 | Diabetic annual review |
| 66AT | Annual diabetic blood test |
| 66AV | Diabetic on insulin+oral treat |
| 66AW | Diabetic foot risk assessment |
| 66AZ | Diabetic monitoring NOS |
| 9OL1 | Attends diabetes monitoring |
| 9OL2 | Refuses diabetes monitoring |
| 9OL4 | Diabetes monitoring 1st letter |
| 9OL5 | Diabetes monitoring 2nd letter |
| 9OL6 | Diabetes monitoring 3rd letter |
| 9OL7 | Diabetes monitor.verbal invite |
| 9OL8 | Diabetes monitor.phone invite |
| 9OL9 | Diabetes monitoring deleted |
| 9OLA | Diabetes monitored |
| 9OLA-1 | Diabetes monitor. check done |
| 9OLD | Diabet pt unsuit dig ret photo |
| C10 | Diabetes mellitus |
| C100 | Diab.mell. - no complication |
| C1000 | Diab.mell.no comp. - juvenile |
| C1000-1 | Insulin dependent diab mellit. |
| C1001 | Diab.mell.no comp. - adult |
| C1001-1 | Maturity onset diabetes |
| C1001-2 | Non-insulin depend.diabet.mell |
| C100z | Diab.mell.no comp. - onset NOS |
| C101 | Diab.mell.with ketoacidosis |
| C1011 | Diab.mell.+ketoacid - adult |
| C101y | Oth specfd diab mel+ketoacidos |
| C101z | Diab.mell.+ketoacid -onset NOS |
| C102 | Diab.mell. + hyperosmolar coma |
| C104 | Diabetic nephropathy with renal manifestation |
| C1041 | Diab.mell.+nephropathy - adult |
| C104-1 | Diabetic nephropathy |
| C104z | Diab.mell.+nephropathy NOS |
| C105 | Diab.mell.+ eye manifestation |
| C1051 | Diab.mell.+eye manif - adult |
| C106 | Diabetes + neuropathy |
| C106-2 | Diab.mell. with neuropathy |
| C1061 | Diab.mell.+neuropathy - adult |
| C106-1 | Diabetic amyotrophy |
| C106-3 | Diabetic mellitus with polyneuropathy |
| C106-99 | Diabetes + neuropathy |
| C106y | Oth specf diab mel+neuro comps |
| C106z | Diab.mell.+neuropathy NOS |
| C1071 | Diab.+periph.circ.dis.-adult |
| C107 | Diab.mell. With peripheral circulatory disorder |
| C107-1 | Diab.mell. With gangrene |
| C107-2 | Diab. With gangrene |
| C1074 | NIDDM periph circulat disord |
| C107z | Diab.+periph.circ.disease NOS |
| C108 | IDDM-Insul depend diabet melit |
| C108-1 | IDDM-Insul depend diabet melit |
| C108-2 | Type 1 diabetes mellitus |
| C108-3 | Type I diabetes mellitus |
| C1085 | Insul depen diab mel+ulcer |
| C1085-1 | TypeI diabetes +ulcer |
| C1085-2 | Insul depen diab mel+ulcer |
| C1088 | Insul dep diab mell-poor contr |
| C1088-1 | Type I diab mell-poor contr |
| C1088-2 | Type 1 diab mell-poor contr |
| C109 | Non-insulin dependent diabetes mellitus |
| C1091 | Non-ins-dp diab mel+ophth comp |
| C109-1 | NIDDM - Non-insulin dependent diabetes mellitus |
| C1091-1 | Type II diab mel+ophth comp |
| C1091-2 | Type 2 diab mel+ophth comp |
| C109-2 | Type 2 diabetes mellitus |
| C1092-2 | Type 2 diab mell neurol comp |
| C109-3 | Type II diabetes mellitus |
| C1094 | Non insulin dependent diab mell with ulcer |
| C1094-1 | Type II diab mell with ulcer |
| C1094-2 | Type 2 diab mell with ulcer |
| C1097 | Non-insul dep diab-poor contr |
| C1097-1 | Type II diab-poor contr |
| C1097-2 | Type 2 diab-poor contr |
| C1099 | Non-insul-dep diab mel no comp |
| C1099-1 | Type II diabetes mellitus without complication |
| C1099-2 | Type 2 diabetes mellitus without complication |
| C109C | Non inslulin dependant diab mell nephropathy |
| C109C-1 | Type II diab mell nephropathy |
| C109C-2 | Type 2 diab mell nephropathy |
| C109J | Insul treated Type 2 diab mell |
| C109J-1 | Insul treated non-insulin dep diab mell |
| C109J-2 | Insul treated Type 2 diab mell |
| C10B | Diabet mel induced by steroids |
| C10B0 | Sterod ind diab mel w/out comp |
| C10E | Insulin dep diabetes mellitus |
| C10E-1 | Insulin dep diabetes mellitus |
| C10E-2 | Insulin dep diabetes mellitus |
| C10E8 | Insul dep diab mell-poor contr |
| C10E8-1 | Type I diab mell-poor contr |
| C10E8-2 | Type 1 diab mell-poor contr |
| C10EC | Type 1 diabetes mellitus with polyneuropathy |
| C10EC-1 | Type I diabetes mellitus with polyneuropathy |
| C10EC-2 | Insulin dependent diabetes mellitus with polyneuropathy |
| C10EK | Type 1 diabetes mellitus with persistent proteinuria |
| C10EL | Type 1 diabetes mellitus with persistent microalbuminuria |
| C10EL-1 | Type I diabetes mellitus with persistent microalbuminuria |
| C10F | Type 2 diabetes mellitus |
| C10F0 | Type 2 diab mell + renal compl |
| C10F0-1 | Type 2 diab mell + renal compl |
| C10F1 | Type 2 diab mell+ophthal comp |
| C10F-1 | Type II diabetes mellitus |
| C10F1-1 | Type II diab mell+ophthal comp |
| C10F2 | Type 2 diab mell + neurol comp |
| C10F2-1 | Type 2 diab mell + neurol comp |
| C10F6 | Type 2 diab mell + retinopathy |
| C10F6-1 | Type II diab mell + retinopathy |
| C10F7 | Type 2 diab mell+poor control |
| C10F7-1 | Type II diab mell+poor control |
| C10F8 | Reaven’s syndrome |
| C10F9 | Type 2 diab mell without comp |
| C10F9-1 | Type II diab mell without comp |
| C10FB | Type 2 diab mell + polyneurop |
| C10FB-1 | Type II diab mell + polyneurop |
| C10FJ | Insul treated Type 2 diab mell |
| C10FJ-1 | Insul treated Type 2 diab mell |
| C10FK | Hyperos non-ket stat typ 2 d m |
| C10FK-1 | Hyperos non-ket stat typ 2 d m |
| C10FL | Type 2 d m + persist proteinur |
| C10FL-1 | Type II d m + persist proteinur |
| C10FM | Type 2 d m + persist microalb |
| C10FM-1 | Type II d m + persist microalb |
| C10FQ | Type 2 d m + exudat maculopath |
| C10FQ-1 | Type II d m + exudat maculopath |
| C10FR | Type 2 dm with gastroparesis |
| C10y | Diab.mell.+other manifestation |
| C10zz | Diab.mell. + unspec comp NOS |

The frozen shoulder Read code list in Table 4 was constructed by two general practitioners. The codes are recorded in the clinical file within CPRD GOLD.

| **Table 4** Table of frozen shoulder Read codes. Note that “..” represents the inclusion of all daughter codes | |
| --- | --- |
| **Read code** | **Detail** |
| N210 | Adhesive capsulitis – shoulder |
| N210-2 | Frozen shoulder |
| EGTON131 | Semi Frozen Shoulder |
| EGTON251 | ? Frozen Right Shoulder |
| N0951 | Shoulder joint stiffness |
| N095A | Stiff shoulder NEC |
| N2120 | Periarthritis of shoulder |
| N21z0 | Capsulitis NOS |
| N0951 | Stiff joint NEC, of the shoulder region |
| N095A | Stiff shoulder NEC |
| N2120 | Periarthritis of shoulder |

The shoulder pain Read code list in Table 5 was obtained from the Prognostic AND Diagnostic Assessment of Shoulder Pain (PANDA-S) study [1] conducted in the Keele University Primary Care Centre Versus Arthritis. The codes are recorded in the clinical file within CPRD GOLD.

| **Table 5** Table of shoulder pain Read codes. Note that “..” represents the inclusion of all daughter codes | |
| --- | --- |
| **Read code** | **Detail** |
| N0511 | Local.primary OA-shoulder regn |
| N0521 | Local.secondary OA-shoulder |
| N0531 | Local.OA unsp.-shoulder region |
| N0541 | Oligoartic OA, unspec-shoulder |
| N05z1 | Osteoarthritis -shoulder joint |
| N05z9 | Osteoarthritis NOS of shoulder |
| N05zA | OA NOS-sternoclavicular joint |
| N05zB | OA NOS-acromioclavicular join |
| N06z1 | Arthropathy NOS-shoulder |
| N0941 | Arthralgia - shoulder |
| N094A | Arthralgia of shoulder |
| N094B | Arthralgia - sternoclav joint |
| N094C | Arthralgia - acromioclav joint |
| N0961 | Other joint sympt.-shoulder |
| N096B | Other symptoms - sternoclav jt |
| N210 | Adhesive capsulitis - shoulde |
| N2110 | Rotator cuff syndrome unspecif |
| N2113 | Supraspinatus tendinitis |
| N211z | Painful arc syndrome |
| N2122 | Subacromial impingement |
| N2124 | Impingement syndr of shoulder |
| N2125 | Shoulder tendonitis |
| N21z2 | Supraspinatus tendonitis |
| N245 | Shoulder pain |
| N2457 | Shoulder pain |
| N210-2 | Frozen shoulder |
| N211z-1 | Painful arc syndrome |
| N245-7 | Shoulder pain |
| N03x0 | Arthr assoc oth dis-shoulder |
| N03x1 | Arthr ass oth dis-sternoclav j |
| N03x2 | Arthr ass oth dis-acromioclv j |
| N0631 | Climacteric arthr.-shoulder |
| N0651 | Unsp.polyarthr.-shoulder |
| N0661 | Unsp.monoarthr.-shoulder |
| N06y1 | Other spec.arthr.-shoulder |
| N0801 | Artic.cart.dis.-shoulder |
| N080B | Artic cart disord oth j-should |
| N0811 | Loose body joint-shoulder |
| N0819 | Loose body in shoulder joint |
| N0841 | Joint contracture-shoulder |
| N084B | Extension contracture-shoulder |
| N084C | Abduction contracture-shoulder |
| N084D | Adduction contracture-shoulder |
| N084E | Int rotat contracture-shoulder |
| N084F | Ext rotat contracture-shoulder |
| N0851 | Joint ankylosis-shoulder |
| N085A | Ankylosis of shoulder joint |
| N08y1 | Oth.joint deran.NEC-shoulder |
| N08z1 | Joint derange.NOS-shoulder |
| N0901 | Joint effusion-shoulder region |
| N090A | Effusion of shoulder |
| N090B | Effusion of sternoclav joint |
| N090C | Effusion of acromioclav joint |
| N0951 | Shoulder joint stiffness |
| N095A | Stiff shoulder NEC |
| N095B | Stiff sternoclavic joint NEC |
| N095C | Stiff acromioclavicular joint NEC |
| N096A | Other symptoms - shoulder |
| N096C | Other symptoms - acromioclav j |
| N0980 | Synov osteochondromat-shoulder |
| N09y1 | Other joint dis.-shoulder |
| N09z1 | Joint disord.NOS-shoulder |
| N211 | Rotator cuff shoulder syndrome |
| N2111 | Calcifying tendinitis shoulder |
| N2114 | Part thickn rotator cuff tear |
| N2115 | Full thickn rotator cuff tear |
| N2118 | Bursitis of shoulder |
| N212 | Other shoulder affections NEC |
| N2120 | Periarthritis of shoulder |
| N2121 | Scapulohumeral fibrositis |
| N212z | Other shoulder affect.NEC NOS |
| N21z0 | Capsulitis NOS |
| NyuAB | [X]Other shoulder lesions |
| S50 | Sprained shoulder |
| S500 | Sprain acromio-clav ligament |
| S501 | Sprain, coraco-clav ligament |
| S504 | Rotator cuff sprain |
| S505 | Sprain subscapularis tendon |
| S507 | Sprain shoulder joint |
| S5070 | Sprain shoulder joint anterior |
| S5071 | Sprain shoulder joint posterior |
| S50w | Other shoulder sprain |
| S50y | Shoulder sprain NOS |
| S5y41 | Sternoclavicular sprain |
| Syu46 | [X]Spr/str oth/un part shl gir |
| N0611 | Traumatic arthropathy of the shoulder region |
| N061A | Traumatic arthropathy of shoulder |
| N0641 | Transient arthropathy of the shoulder region |
| N064A | Transient arthropathy of shoulder |
| N0661 | Unspecified monoarthritis of the shoulder region |
| N06z1 | Arthropathy NOS, of the shoulder region |
| N0831 | Recurrent joint dislocation, of the shoulder region |
| N083A | Recurrent dislocation of shoulder - anterior |
| N083B | Recurrent dislocation of shoulder - posterior |
| N083C | Recurrent subluxation of shoulder - anterior |
| N083D | Recurrent subluxation of shoulder - posterior |
| N083E | Recurrent dislocation of shoulder - inferior |
| N083F | Recurrent subluxation of shoulder - inferior |
| N083G | Recurrent dislocation of shoulder - anterior |
| N083H | Recurrent subluxation of shoulder - anterior |
| N083J | Recurrent dislocation of shoulder - multidirectional |
| N083K | Recurrent subluxation of shoulder - multidirectional |
| N083L | Habitual dislocation of the shoulder |
| N083M | Habitual subluxation of the shoulder |
| N0878 | Snapping shoulder |
| N08y1 | Other joint derangement NEC, of the shoulder region |
| N08z1 | Joint derangement NOS, of the shoulder region |
| N0911 | Haemarthrosis of the shoulder region |
| N091A | Haemarthrosis of shoulder |
| N092B | Villonodular synovitis of sternoclavicular joint |
| N092C | Villonodular synovitis of acromioclavicular joint |
| N0951 | Stiff joint NEC, of the shoulder region |
| N095A | Stiff shoulder NEC |
| N09y1 | Other specified joint disorders of the shoulder region |
| N09z1 | Joint disorder NOS, of shoulder region |
| N2120 | Periarthritis of shoulder |
| S5Q6 | Injury of tendon of the rotator cuff of shoulder |
| SD28 | Multiple superficial injuries of shoulder and upper arm |
| SD2y | Superficial injury shoulder/upper arm NOS, without infection |
| SD2y0 | Superficial injury shoulder NOS |
| SD2yz | Superficial injury shoulder/upper arm, without infection |
|  | NOS |
| SK12 | Other shoulder and upper arm injuries |
| SK122 | Other shoulder injuries |
| SK12z | Other shoulder and upper arm injury NOS |
| Syu4 | [X]Injuries to the shoulder and upper arm |
| Syu40 | [X]Other superficial injuries of shoulder and upper arm |
| Syu4D | [X]Other specified injuries of shoulder and upper arm |
| Syu4E | [X]Unspecified injury of shoulder and upper arm |
| 1M02 | Shoulder joint painful on movement |
| 1M03 | Shoulder joint painful on external rotation |
| N2457 | Shoulder pain |
| 7NC3 | [SO]Ligament of shoulder or elbow |
| 7NC30 | [SO]Ligament of sternoclavicular joint |
| 7NC31 | [SO]Ligament of acromio-clavicular joint |
| 7NC32 | [SO]Coraco-clavicular ligament |
| 7NC33 | [SO]Coraco-acromial ligament |
| 7NC34 | [SO]Gleno-humeral ligament |
| 7NC35 | [SO]Glenoid labrum |
| 7NC36 | [SO]Ligament of elbow joint |
| 7NC37 | [SO]Ligament of superior radio-ulnar joint |
| 7NC38 | [SO]Annular ligament |
| 7NC39 | [SO]Ligament of inferior radio ulnar joint |
| S50 | Sprain of shoulder and upper arm |
| S500 | Sprain, acromio-clavicular ligament |
| S501 | Sprain, coraco-clavicular ligament |
| S502 | Coracohumeral sprain |
| S503 | Sprain, infraspinatu tendon |
| S504 | Rotator cuff sprain |
| S505 | Sprain, subscapularis tendon |
| S506 | Sprain, supraspinatus tendon |
| S507 | Sprain, shoulder joint |
| S5070 | Sprain, shoulder joint, anterior |
| S5071 | Sprain, shoulder joint, posterior |
| S508 | Sprain, biceps tendon |
| S509 | Sprain, long head of biceps tendon |
| S50A | Sprain, triceps tendon |
| S50X | Sprain and strain of other and unspecified parts of shoulder girdle |
| S50w | Other shoulder sprain |
| S50x | Other upper arm sprain |
| S50y | Shoulder sprain NOS |
| S50z | Upper arm sprain NOS |
| N2124 | Impingement syndrome of shoulder |
| 7N82 | [SO]Muscle of shoulder or upper arm |
| 7N820 | [SO]Deltoid |
| 7N821 | [SO]Rotator cuff |
| 7N823 | [SO]Biceps brachii |
| 7N827 | [SO]Supraspinatus |
| 7N828 | [SO]Flexor of upper arm |
| 7N829 | [SO]Extensor of upper arm |
| 7N82y | [SO]Specified muscle of shoulder or upper arm NEC |
| 7N82z | [SO]Muscle of shoulder or upper arm NEC |
| 7NAD | [SO]Joint of shoulder girdle or arm |
| 7NAD0 | [SO]Sternoclavicular joint |
| 7NAD1 | [SO]Acromioclavicular joint |
| 7NAD2 | [SO]Glenohumeral joint |
| 7NAD3 | [SO]Shoulder joint |
| 7NADz | [SO]Joint of shoulder girdle or arm NEC |
| N211 | Rotator cuff shoulder syndrome and allied disorders |
| N2110 | Rotator cuff syndrome, unspecified |
| N2111 | Calcifying tendinitis of the shoulder |
| N2112 | Bicipital tenosynovitis |
| N2113 | Supraspinatus tendinitis |
| N2114 | Partial thickness rotator cuff tear |
| N2115 | Full thickness rotator cuff tear |
| N2116 | Subacromial bursitis |
| N2117 | Subdeltoid bursitis |
| N2118 | Bursitis of shoulder |
| N211z | Rotator cuff syndrome NOS |

The hypertension Read code list in Table 6 was obtained from another study (Blagojevic-Bucknall et al., 2017 [2]) conducted in the Primary Care Centre Versus Arthritis. The codes are recorded in the clinical file within CPRD GOLD.

| **Table 6** Table of hypertension Read codes. Note that “..” represents the inclusion of all daughter codes | |
| --- | --- |
| **Read code** | **Detail** |
| G24z100 | Hypertension secondary to drug |
| G202.00 | Systolic hypertension |
| G21z.00 | Hypertensive heart disease NOS |
| 9OI..00 | Hypertension monitoring admin. |
| G24z000 | Secondary renovascular hypertension NOS |
| G210.00 | Malignant hypertensive heart disease |
| 9OI1.00 | Attends hypertension monitor. |
| G22z.11 | Renal hypertension |
| 9OIA.11 | Hypertension monitored |
| G241z00 | Secondary benign hypertension NOS |
| G203.00 | Diastolic hypertension |
| 9OI..11 | Hypertension clinic admin. |
| G20..00 | Essential hypertension |
| 662O.00 | On treatment for hypertension |
| 8HT5.00 | Referral to hypertension clinic |
| G201.00 | Benign essential hypertension |
| 8CR4.00 | Hypertension clinical management plan |
| 662..12 | Hypertension monitoring |
| G240z00 | Secondary malignant hypertension NOS |
| 6629 | Hypertension:follow-up default |
| G240000 | Secondary malignant renovascular hypertension |
| G22..00 | Hypertensive renal disease |
| G222.00 | Hypertensive renal disease with renal failure |
| Gyu2100 | [X]Hypertension secondary to other renal disorders |
| 662P.00 | Hypertension monitoring |
| 8I3N.00 | Hypertension treatment refused |
| 662c.00 | Hypertension six month review |
| G20z.00 | Essential hypertension NOS |
| G2...11 | BP - hypertensive disease |
| G20..11 | High blood pressure |
| 662G.00 | Hypertensive treatm.changed |
| 9N1y200 | Seen in hypertension clinic |
| 662H.00 | Hypertension treatm.stopped |
| G244.00 | Hypertension secondary to endocrine disorders |
| 67H8.00 | Lifestyle advice regarding hypertension |
| 9h31.00 | Excepted from hypertension qual indicators: Patient unsuit |
| 6627 | Good hypertension control |
| G24zz00 | Secondary hypertension NOS |
| 9OI2.00 | Refuses hypertension monitor. |
| 9OI4.00 | Hypertens.monitor.1st letter |
| 6628 | Poor hypertension control |
| G240.00 | Secondary malignant hypertension |
| 662b.00 | Moderate hypertension control |
| 662d.00 | Hypertension annual review |
| G220.00 | Malignant hypertensive renal disease |
| 14A2.00 | H/O: hypertension |
| G20z.11 | Hypertension NOS |
| G232.00 | Hypertensive heart&renal dis wth (congestive) heart failure |
| G21zz00 | Hypertensive heart disease NOS |
| G234.00 | Hyperten heart&renal dis+both(congestv)heart and renal fail |
| 662F.00 | Hypertension treatm. started |
| Gyu2.00 | [X]Hypertensive diseases |
| G210000 | Malignant hypertensive heart disease without CCF |
| G21z011 | Cardiomegaly - hypertensive |
| 9OIA.00 | Hypertension monitor.chck done |
| G200.00 | Malignant essential hypertension |
| 9h32.00 | Excepted from hypertension qual indicators: Informed dissent |
| G2...00 | Hypertensive disease |
| G24z.00 | Secondary hypertension NOS |
| 9OI6.00 | Hypertens.monitor 3rd letter |
| G24..00 | Secondary hypertension |
| 9N03.00 | Seen in hypertension clinic |
| G241.00 | Secondary benign hypertension |
| G221.00 | Benign hypertensive renal disease |
| G211.00 | Benign hypertensive heart disease |
| G2z..00 | Hypertensive disease NOS |
| 9OI5.00 | Hypertens.monitor 2nd letter |
| 662q.00 | Trial reduction of antihypertensive therapy |
| 9OI7.00 | Hypertens.monitor verbal inv. |
| G241000 | Secondary benign renovascular hypertension |
| G21..00 | Hypertensive heart disease |
| 9OI8.00 | Hypertens.monitor phone invite |
| G2y..00 | Other specified hypertensive disease |
| G211100 | Benign hypertensive heart disease with CCF |
| G22z.00 | Hypertensive renal disease NOS |

The hyperlipidaemia Read code list in Table 7 was obtained from another study (Blagojevic-Bucknall et al., 2017 [2]) conducted in the Primary Care Centre Versus Arthritis. The codes are recorded in the clinical file within CPRD GOLD.

| **Table 7** Table of hyperlipidaemia Read codes. Note that “..” represents the inclusion of all daughter codes | |
| --- | --- |
| **Read code** | **Detail** |
| C320100 | Hyperbetalipoproteinaemia |
| 9N0J.00 | Seen in cholesterol clinic |
| 8I3J.00 | Lipid lowering therapy declined |
| Cyu8D00 | [X]Other hyperlipidaemia |
| C320200 | Hyperlipidaemia, group A |
| 1442 | H/O: raised blood lipids |
| C321.00 | Pure hyperglyceridaemia |
| ZV65317 | [V]Dietary surveillance in hypercholesterolaemia |
| 8B28.00 | Lipid lowering therapy |
| C321000 | Hypertriglyceridaemia |
| 9Oc3.00 | Lipid disorder monitoring second letter |
| 9Oc..00 | Lipid disorder monitoring administration |
| 8BAG.00 | Cholesterol reduction programme |
| C320300 | Low |
| 66X..00 | Lipid disorder monitoring |
| 8B6A.00 | Statin prophylaxis |
| C320500 | Familial defective apolipoprotein B-100 |
| C320y00 | Other specified pure hypercholesterolaemia |
| 8I76.00 | Statin not tolerated |
| C322.11 | Fredrickson type IIb lipidaemia |
| C322.12 | Fredrickson type III lipidaemia |
| 8CR3.00 | Hyperlipidaemia clinical management plan |
| 8BAG200 | Cholesterol reduction program - declined |
| C324.00 | Hyperlipidaemia NOS |
| C325000 | High density lipoid deficiency |
| C327z00 | Lipidoses NOS |
| 44P3.00 | Serum cholesterol raised |
| C323.12 | Fredrickson type I lipaemia |
| 8I3C.00 | Statin declined |
| C325.00 | Lipoprotein deficiencies |
| C322.00 | Mixed hyperlipidaemia |
| C328.00 | Dyslipidaemia |
| C320.12 | Fredrickson type IIa lipidaemia |
| C320.13 | Low density lipoproteinaemia |
| C320.11 | Familial hypercholesterolaemia |
| 8BG2.00 | Lipid lowering therapy indicated |
| 8BAG000 | Cholesterol reduction programme - invited |
| C320z00 | Pure hypercholesterolaemia NOS |
| C320000 | Familial hypercholesterolaemia |
| 44P4.00 | Serum cholesterol very high |
| C321.11 | Fredrickson type IV lipidaemia |
| C321.12 | Very low density lipoprotinaemia |
| 9Oc0.00 | Attends lipid disorder monitoring |
| 8HT1.00 | Referral to lipid clinic |
| C320.00 | Pure hypercholesterolaemia |
| 9N0I.00 | Seen in lipid clinic |
| 9N4K.00 | DNA - Did not attend cholesterol clinic |
| 8BL1.00 | Patient on maximal tolerated lipid lowering therapy |
| ZC2CJ00 | Dietary advice for hyperlipidaemia |
| 8BAG100 | Cholesterol reduction program - attended |

The thyroid dysfunction Read code list in Table 8 was constructed by a general practitioner. The codes are recorded in the clinical file within CPRD GOLD.

| **Table 8 Table of thyroid dysfunction Read codes. Note that “..” represents the inclusion of all daughter codes** | |
| --- | --- |
| **Read code** | **Detail** |
| C046.00 | Autoimmune myxoedema |
| A175.00 | Tuberculosis of thyroid gland |
| 9O39100 | Thyroid monitoring SMS text message first invitation |
| C02y200 | Thyrotoxicosis factitia |
| 7113y00 | Other specified other operation on thyroid gland |
| C043z00 | Iatrogenic hypothyroidism NOS |
| C051.11 | De Quervain’s thyroiditis |
| C043200 | Hypothyroidism resulting from resorcinol |
| 1433 | H/O: thyroid disorder NOS |
| C04z100 | Myxoedema coma |
| 711z.00 | Thyroid gland and parathyroid gland operations NOS |
| BB5fz00 | [M]Thyroid adenoma or adenocarcinoma NOS |
| C000.13 | Thyroid nodule |
| C03y100 | Congenital hypothyroidism without goitre |
| U602100 | [X]Thyroid horms + substits caus adverse eff in therap use |
| C02yz00 | Thyrotoxicosis of other specified origin NOS |
| PK25z00 | Anomaly of thyroid gland NEC NOS |
| C02..00 | Thyrotoxicosis |
| C052.11 | Autoimmune thyroiditis |
| C02y000 | Thyrotoxicosis of other specified origin with no crisis |
| C050.00 | Acute thyroiditis |
| F395300 | Myopathy due to myxoedema |
| 5A12.00 | Thyroid tumour/metast irradiat |
| 4423 | Thyroid hormone tests low |
| F4G2000 | Thyrotoxic exophthalmos |
| C044.00 | Postinfectious hypothyroidism |
| 7113z00 | Other operation on thyroid gland NOS |
| ByuB.00 | [X]Malignant neoplasm of thyroid and other endocrine glands |
| C03z.00 | Congenital hypothyroidism NOS |
| 7110000 | Total thyroidectomy |
| C03..00 | Congenital hypothyroidism |
| 7N10000 | [SO]Thyroid gland |
| 7111y00 | Other specified operation on aberrant thyroid tissue |
| Cyu1400 | [X]Other chronic thyroiditis |
| C062.00 | Thyroid cyst |
| C04z.12 | Thyroid insufficiency |
| C03y.00 | Other specified congenital hypothyroidism |
| C022.00 | Toxic multinodular goitre |
| TJ27000 | Adverse reaction to liothyronine sodium |
| C024z00 | Thyrotoxicosis from ectopic thyroid nodule NOS |
| C024000 | Thyrotoxicosis from ectopic thyroid nodule with no crisis |
| C00z.00 | Goitre NOS |
| L181z00 | Thyroid dysfunction in pregnancy/childbirth/puerperium |
|  | NOS |
| 9O39200 | Thyroid monitoring SMS text message 2nd invitation |
| C05y.00 | Other and unspecified chronic thyroiditis |
| 8B71.00 | Iodine-goitre prophylaxis |
| C011.00 | Nontoxic multinodular goitre |
| C03z.11 | Congenital thyroid insufficiency |
| 1431 | H/O: hyperthyroidism |
| C061.00 | Dyshormonogenic goitre |
| C041z00 | Postablative hypothyroidism NOS |
| C052.00 | Chronic lymphocytic thyroiditis |
| C052.12 | Hashimoto’s disease |
| 7110300 | Lobectomy of thyroid gland NEC |
| 66B4.00 | Thyroid eye disease |
| C04z000 | Premature puberty due to hypothyroidism |
| Cyu1300 | [X]Other thyrotoxicosis |
| 212P.00 | Hyperthyroidism resolved |
| TJ27200 | Adverse reaction to thyroglobulin |
| C06y000 | Thyroid-binding globulin abnormality |
| C03y000 | Congenital hypothyroidism with diffuse goitre |
| C021000 | Toxic uninodular goitre with no crisis |
| 66B..00 | Thyroid disease monitoring |
| C02z.00 | Thyrotoxicosis without mention of goitre or other cause |
| B8yy000 | Carcinoma in situ of thyroid gland |
| PK25.00 | Anomalies of thyroid gland NEC |
| C000.12 | Substernal thyroid goitre |
| 1431.11 | H/O: thyrotoxicosis |
| Cyu1100 | [X]Other sp cified hypothyroidism |
| C020200 | Thyroid-associated dermopathy |
| FyuBD00 | [X]Dysthyroid exophthalmos |
| C02y100 | Thyrotoxicosis of other specified origin with crisis |
| Cyu1600 | [X]Iodine-deficiency-related (endemic) goitre, unspecified |
| 9h71.00 | Excepted from thyroid quality indicators: Patient unsuitable |
| AC22.00 | Thyroid echinococcus granulosus |
| C047.00 | Subclinical hypothyroidism |
| 7113100 | Biopsy of lesion of thyroid gland |
| C134300 | TSH - thyroid-stimulating hormone deficiency |
| 1432 | H/O: hypothyroidism |
| F144100 | Cerebellar ataxia due to myxoedema |
| C0A1.00 | Congenital iodine-deficiency syndrome, myxoedematous type |
| C0A5.00 | Subclinical iodine-deficiency hypothyroidism |
| 7N10100 | [SO]Aberrant thyroid tissue |
| C020.00 | Toxic diffuse goitre |
| C025.00 | Subclinical hyperthyroidism |
| G557500 | Thyrotoxic heart disease |
| Q44V.00 | Neonatal goitre, not elsewhere classified |
| 711..00 | Thyroid gland and parathyroid gland operations |
| C000.14 | Colloid goitre |
| C000.11 | Retrosternal thyroid goitre |
| 9O39300 | Thyroid monitoring SMS text message third invitation |
| 5A11.00 | Thyroid gland ablat - irradiat |
| 4422 | Thyroid hormone tests high |
| C043000 | Hypothyroidism resulting from para-aminosalicylic acid |
| C023z00 | Toxic nodular goitre NOS |
| C0...00 | Disorders of thyroid gland |
| 7110200 | Hemithyroidectomy |
| 66BA.00 | Thyroid dis.treatment stopped |
| 7110z00 | Thyroidectomy NOS |
| C04z.00 | Hypothyroidism NOS |
| Qyu6400 | [X]Other transitory neonatal disorders/thyroid function,NEC |
| L181200 | Thyroid dysfunction in puerperium - baby delivered |
| Q443.00 | Neonatal thyrotoxicosis |
| 442G.00 | Thyroid hormone tests abnormal |
| C0AX.00 | Iodine-deficiency-related (endemic) goitre, unspecified |
| C05z.00 | Thyroiditis NOS |
| C010.00 | Nontoxic uninodular goitre |
| 687H.00 | Congenital hypothyroidism screening related finding |
| SL27.00 | Thyroid hormone and thyroid derivatives poisoning |
| TJ28z00 | Adverse reaction to antithyroid agents NOS |
| 7113200 | Incision of lesion of thyroid gland |
| TJ27.00 | Adverse reaction to thyroid and thyroid derivatives |
| 66B8.00 | Thyroid dis.treatment changed |
| 9Oj..00 | Hypothyroidism monitoring administration |
| 9Oj1.00 | Hypothyroidism monitoring second letter |
| 9Oj3.00 | Hypothyroidism monitoring verbal invite |
| 66B5.00 | Thyroid symptom change |
| C063000 | Thyroid haemorrhage |
| 9Oj4.00 | Hypothyroidism monitoring telephone invitation |
| 711..12 | Thyroid gland operations |
| F381600 | Myasthenic syndrome due to thyrotoxicosis |
| C063.00 | Thyroid haemorrhage and infarction |
| C0A4.00 | Iodine-deficiency-related multinodular (endemic) goitre |
| 7110400 | Isthmectomy of thyroid gland |
| SL27300 | Thyroglobulin poisoning |
| C023000 | Toxic nodular goitre unspecified with no crisis |
| C063100 | Thyroid infarction |
| C01z.11 | Adenomatous goitre |
| C0...11 | Struma - goitre |
| 66BA.11 | Thyroxine Rx stopped |
| 8CR5.00 | Hypothyroidism clinical management plan |
| L181000 | Thyroid dysfunction - unspec whether in pregnancy/puerperium |
| C045.00 | Acquired atrophy of thyroid |
| Cyu1.00 | [X]Disorders of thyroid gland |
| C050200 | Abscess of thyroid |
| 66B3.00 | Inactive thyroid disease |
| C053.00 | Chronic fibrous thyroiditis |
| C05y400 | Chronic thyroiditis with transient thyrotoxicosis |
| F395400 | Myopathy due to thyrotoxicosis |
| Cyu1200 | [X]Other specified nontoxic goitre |
| 6762 | Education about thyroid disease in pregnancy |
| 22H3.00 | O/E - thyroid swelling -bilat. |
| 7113300 | Exploration of thyroid gland |
| C0A3.00 | Iodine-deficiency-related diffuse (endemic) goitre |
| C06yz00 | Other specified thyroid disorder NOS |
| L181400 | Thyroid dysfunction in puerperium- baby previously delivered |
| B53..00 | Malignant neoplasm of thyroid gland |
| C020100 | Toxic diffuse goitre with crisis |
| C06z.00 | Thyroid disorder NOS |
| 442I.00 | Thyroid function tests abnormal |
| C00..00 | Simple and unspecified goitre |
| 7113000 | Excision of lesion of thyroid gland |
| C050z00 | Acute thyroiditis NOS |
| C02z100 | Thyrotoxicosis without mention of goitre, cause with crisis |
| R145.00 | [D]Thyroid function test abnormal |
| C041000 | Irradiation hypothyroidism |
| PK25011 | Retrosternal thyroid gland |
| C06y100 | Thyroid atrophy |
| C06..00 | Other disorders of thyroid |
| 9h72.00 | Excepted from thyroid quality indicators: Informed dissent |
| C023100 | Toxic nodular goitre unspecified with crisis |
| SL27z00 | Thyroid hormone and thyroid derivative poisoning NOS |
| C021.00 | Toxic uninodular goitre |
| C023.00 | Toxic nodular goitre unspecified |
| C053.11 | Riedel’s thyroiditis |
| R145000 | [D]Thyroid scan abnormal |
| C021z00 | Toxic uninodular goitre NOS |
| TJ28.00 | Adverse reaction to antithyroid agents |
| R145z00 | [D]Thyroid function tests abnormal NOS |
| C04y.00 | Other acquired hypothyroidism |
| 66B9.00 | Thyroid dis.treatment started |
| C04..13 | Hypothyroidism |
| C04..12 | Thyroid deficiency |
| C04..11 | Myxoedema |
| L181100 | Thyroid dysfunction during pregnancy - baby delivered |
| C04z.13 | Hypothyroid goitre, acquired |
| C04z.11 | Pretibial myxoedema - hypothyroid |
| C01z.00 | Nontoxic nodular goitre NOS |
| Q433700 | Neonatal jaundice with congenital hypothyroidism |
| 9Oj0.00 | Hypothyroidism monitoring first letter |
| 9Oj2.00 | Hypothyroidism monitoring third letter |
| 66B2.00 | Follow-up thyroid assessment |
| C02..11 | Hyperthyroidism |
| C02..12 | Toxic goitre |
| 66B6.00 | Thyroid drug side effects |
| C00z.11 | Thyroid enlargement |
| 7111000 | Excision of substernal thyroid tissue |
| 66BB.00 | Hypothyroidism annual review |
| U602200 | [X]Antithyroid drugs caus adverse effects in therapeut use |
| C040.00 | Postsurgical hypothyroidism |
| 7111z00 | Operation on aberrant thyroid tissue NOS |
| 1JM..00 | Suspected hypothyroidism |
| C022100 | Toxic multinodular goitre with crisis |
| B7G..11 | Adenoma of thyroid gland |
| C02y.11 | Factitia thyrotoxicosis |
| Cyu1500 | [X]Other specified disorders of thyroid |
| C020000 | Toxic diffuse goitre with no crisis |
| Cyu4J00 | [X]Disorders of thyroid gland in diseases CE |
| N220411 | De Quervain’s disease |
| C02z000 | Thyrotoxicosis without mention of goitre or cause no crisis |
| C02zz00 | Thyrotoxicosis NOS |
| 8BPG.00 | Thyroid stimulating hormone suppression therapy |
| C054.00 | Iatrogenic thyroiditis |
| 22H2.00 | O/E - thyroid swelling -unilat |
| 7L1Z400 | Oral delivery of radiotherapy for thyroid ablation |
| 711y.00 | Thyroid gland or parathyroid gland operations OS |
| 7110600 | Thyroidectomy NEC |
| 9N4T.00 | DNA - Did not attend hyperthyroidism clinic |
| 7110 | Thyroidectomy operations |
| 66BZ.00 | Thyroid disease monitoring NOS |
| C022000 | Toxic multinodular goitre with no crisis |
| PK25000 | Aberrant thyroid gland |
| PK25100 | Congenital absence of thyroid gland |
| BB5f.00 | [M]Thyroid adenoma and adenocarcinoma |
| 7110.11 | Excision of thyroid gland operations |
| R145100 | [D]Thyroid uptake abnormal |
| 66B7.00 | Thyroid-dubious diagn.criteria |
| 44qV000 | Congenital hypothyroidism screening, borderline result |
| C06y.00 | Other specified thyroid disorders |
| 143..11 | H/O: thyroid disorder |
| C051.00 | Subacute thyroiditis |
| 7110500 | Partial thyroidectomy NEC |
| L181500 | Postpartum thyroiditis |
| C050000 | Acute nonsuppurative thyroiditis |
| TJ27100 | Adverse reaction to thyroxine sodium |
| C000.00 | Simple goitre |
| B924000 | Neoplasm of uncertain behaviour of thyroid gland |
| U602113 | [X] Adverse reaction to levothyroxine sodium |
| 7111 | Operations on aberrant thyroid tissue |
| C041.00 | Other postablative hypothyroidism |
| 7111100 | Excision of sublingual thyroid tissue |
| 66B9.11 | Thyroxine Rx started |
| C05..00 | Thyroiditis |
| 7110y00 | Other specified thyroidectomy |
| SL28.00 | Antithyroid agent poisoning |
| ZV10y15 | [V]Personal history of malignant neoplasm of thyroid |
| 7110111 | Bilateral subtotal thyroidectomy |
| U602211 | [X] Adverse reaction to antithyroid agents |
| C040.11 | Post ablative hypothyroidism |
| 442C.00 | Thyroid horm tests borderline |
| B7G..00 | Benign neoplasm of thyroid gland |
| C02y.00 | Thyrotoxicosis of other specified origin |
| 9O39.00 | Thyroid monitoring call |
| C042.00 | Iodine hypothyroidism |
| L181.00 | Thyroid dysfunction in pregnancy/childbirth/puerperium |
| 22H4.00 | O/E - thyroid lump |
| C043.00 | Other iatrogenic hypothyroidism |
| 7110100 | Subtotal thyroidectomy |
| 7113 | Other operations on thyroid gland |
| 1JM0.00 | Suspected congenital hypothyroidism |
| F381400 | Myasthenic syndrome due to hypothyroidism |
| L181300 | Thyroid dysfunction in pregnancy - baby not yet delivered |
| 9h7..00 | Exception reporting: thyroid quality indicators |
| F11x500 | Cerebral degeneration due to myxoedema |
| TJ27z00 | Adverse reaction to thyroid and thyroid derivatives NOS |
| C020z00 | Toxic diffuse goitre NOS |
| C022z00 | Toxic multinodular goitre NOS |
| C01..00 | Nontoxic nodular goitre |
| SL28z00 | Antithyroid agent poisoning NOS |
| C02y300 | Thyroid crisis |
| C024.00 | Thyrotoxicosis from ectopic thyroid nodule |
| C04..00 | Acquired hypothyroidism |
| C050100 | Acute suppurative thyroiditis |

| **Table 9** Table describing the source of ethnicity, deprivation, smoking, alcohol, weight, height data within CPRD | | | |
| --- | --- | --- | --- |
| **Variable** | **Source** | **enttype** | **Data column** |
| Ethnicity | HES | n/a | gen_ethnicity |
| Deprivation (IMD) | IMD | n/a | imd2015_5 |
| Weight | Additional | 13 | data1 |
|  | CPRD files | |  |
| Height | Additional | 14 | data1 |
|  | CPRD files | |  |

**References**

[1] Wynne-Jones G, Myers H, et al. Predicting pain and function outcomes in people consulting with shoulder pain: the PANDA-S clinical cohort and qualitative study protocol. BMJ Open 2021;11:e052758. doi: 10.1136/bmjopen-2021-052758

[2] Blagojevic-Bucknall M, C. Mallen, et al. Gout as a consequence of sleep apnoea: a matched cohort study. Rheumatology, 2017;56(2):183 doi: 10.1002/art.40662
