# Supplementary material for: Type 2 diabetes, metabolic health, and the development of frozen shoulder: a cohort study in UK electronic health records
Source: BMC Musculoskelet Disord. 2025 May 14;26:471. doi: 10.1186/s12891-025-08672-2 (PMC12080057; doi:10.1186/s12891-025-08672-2)
Supplement: Supplementary file 2 — Supplementary Material 2 [file 12891_2025_8672_MOESM2_ESM.docx]

**Appendix B – Categorical variable levels**

Factor levels of categorical variables containing low cell counts (<3%) were collapsed. Below are the original and collapsed factor levels for categorical variables.

**Ethnicity original:** Bangladeshi, black African, black Caribbean, black–other, Chinese, Indian, mixed, other Asian, other, Pakistani, missing, white.

**Ethnicity after collapsing levels:** white, not white, missing.

**Alcohol original:** currently drink alcohol, never drunk alcohol, have quit drinking alcohol, missing.

**Alcohol after collapsing levels:** do drink/have previously drunk alcohol, never drunk alcohol, missing.

All other categorical variables were left with their original categories. The categories are given below:

**Gender:** male, female.

**Deprivation:** least deprived IMD quintile, 2nd least deprived quintile, 3rd least deprived quintile, 4th least deprived quintile, most deprived quintile.

**Smoking:** current smoker, ex-smoker, never smoked, missing.

**Thyroid dysfunction:** diagnosed, not diagnosed.

**Hyperlipidaemia:** diagnosed, not diagnosed.

**Hypertension:** diagnosed, not diagnosed.

**Obesity:** obese, not obese, missing.

**Type 2 diabetes:** diagnosed, not diagnosed.

**Frozen shoulder:** diagnosed, not diagnosed.
