# Supplementary material for: Type 2 diabetes, metabolic health, and the development of frozen shoulder: a cohort study in UK electronic health records
Source: BMC Musculoskelet Disord. 2025 May 14;26:471. doi: 10.1186/s12891-025-08672-2 (PMC12080057; doi:10.1186/s12891-025-08672-2)
Supplement: Supplementary file 3 — Supplementary Material 3 [file 12891_2025_8672_MOESM3_ESM.docx]

**Appendix C**

**Schoenfeld Residual Plots**


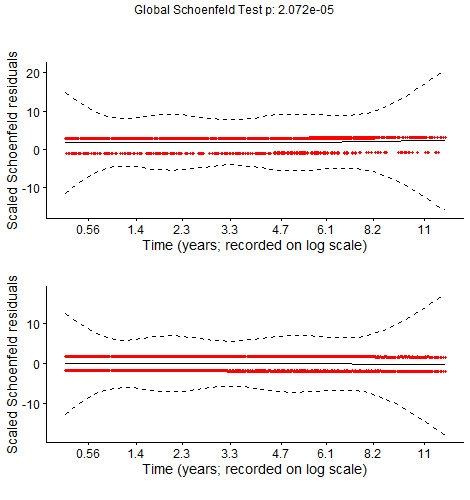


**Fig C.1** A scaled Schoenfeld residual plot with an added smoothing spline and 95% confidence bands, for the natural direct effect coefficient in the Cox model


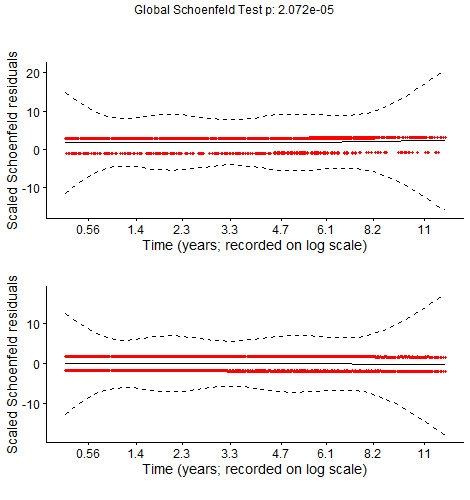


**Fig C.2** A scaled Schoenfeld residual plot with an added smoothing spline and 95% confidence bands, for the natural indirect effect coefficient in the Cox model
