# Supplementary material for: Type 2 diabetes, metabolic health, and the development of frozen shoulder: a cohort study in UK electronic health records
Source: BMC Musculoskelet Disord. 2025 May 14;26:471. doi: 10.1186/s12891-025-08672-2 (PMC12080057; doi:10.1186/s12891-025-08672-2)
Supplement: Supplementary file 4 — Supplementary Material 4 [file 12891_2025_8672_MOESM4_ESM.docx]

**Appendix D**


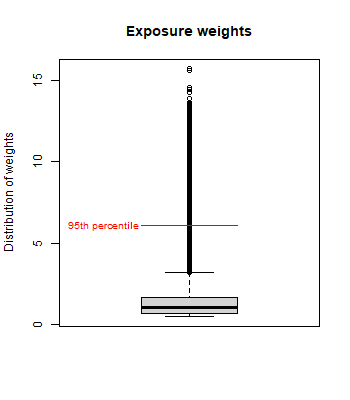


**Fig D.1** Box plot of exposure weights, $W_{i}^{X}$


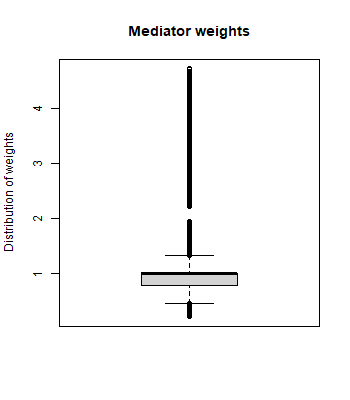


**Fig D.2** Box plot of mediator weights, $W_{i}^{M}$


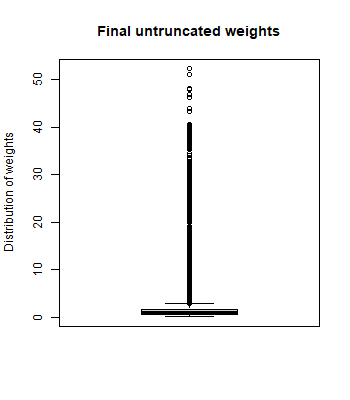


**Fig D.3** Box plot of final untruncated Cox model weights, $W_{i},\text{untruncated}$


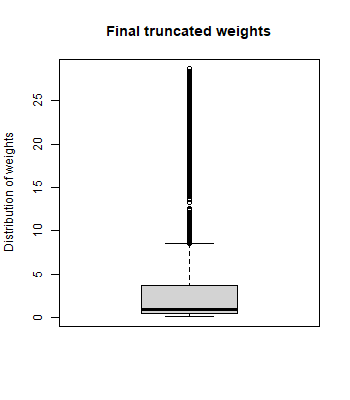


**Fig D.4** Box plot of final truncated Cox model weights, $W_{i},\text{truncated}$
